# Displaced sigmoid notch fracture and higher patient age are associated with distal radioulnar joint subluxation

**DOI:** 10.55730/1300-0144.5801

**Published:** 2024-07-07

**Authors:** Saygın KAMACI, Melih ORAL, Engin Türkay YILMAZ, Taha AKSOY, Barış KAFA, Mazhar TOKGÖZOĞLU

**Affiliations:** 1 Department of Orthopedics and Traumatology, Faculty of Medicine, Hacettepe University, Ankara, Turkiye; 2 Department of Orthopedics and Traumatology, Gülhane Training and Research Hospital, University of Health Sciences, Ankara, Turkiye

**Keywords:** Distal radioulnar joint instability, sigmoid notch fracture, ulna styloid fracture

## Abstract

**Background/aim:**

Distal radius fractures (DRFs) are frequently associated with distal radioulnar joint (DRUJ) instability. The purpose of this study is to evaluate the effect of the sigmoid notch and ulna styloid fracture types on DRUJ subluxation following closed reduction and casting of DRFs via calculating radioulnar ratio (RUR) on postreduction computed tomography (CT) images.

**Materials and methods:**

In our study, postreduction CT images of 202 patients with distal radius fractures were evaluated retrospectively. CT images were evaluated for RUR, sigmoid notch fracture, and ulna styloid types. Sigmoid notch fractures were classified as nondisplaced in the sigmoid notch fractures (NDS) and displaced sigmoid notch (DS) fractures; ulna styloid fractures were grouped as the proximal half ulna styloid (PHUS) and distal half ulna styloid (DHUS) fractures.

**Results:**

The mean age of Rozental type 3b (62.8 years) was significantly higher among others. The mean RUR value was significantly higher in Rozental type 3a in compared to type 1a and type 2 fractures. PHUS fractures were more common with DS fractures than DHUS fractures.

**Conclusion:**

DS fractures and higher patient age are associated with DRUJ subluxation on postreduction CT images following DRFs. DS fractures are seen more commonly with PHUS fractures than DHUS. Patients with PHUS should be carefully assessed for sigmoid notch fractures and DRUJ congruency. These findings could be helpful for preoperative decision making in the treatment of DRFs.

## 1. Introduction

Fractures of the distal radius (DRFs) stand as a frequent occurrence, with their incidence on the rise [1]. Debates persist around the most effective treatment, and a prerequisite for navigating this uncertainty is an in-depth analysis through initial imaging. This allows for a comprehensive understanding of fracture morphology, stability, and any accompanying issues. Distal radioulnar joint (DRUJ) instability is known as a problem that causes pain and disability following DRFs. DRUJ stability is provided by bony and ligamentous anatomy. Structures that contribute to rotatory and axial stability of the forearm include osseous anatomy of the radius and ulna, triangular fibrocartilage complex (TFCC), pronator quadratus, extensor retinaculum, intraosseous membrane, and annular ligament of the elbow.

TFCC is the primary stabilizing structure of the DRUJ. TFCC injury is common and diagnosed with 60%–84% of DRFs [2]. DRUJ instability following TFCC injury is a multifactorial condition seen at a rate of 3%–37% following DRFs [3]. Thus, predicting a potential DRUJ instability is crucial. Various radiological stigmas for DRUJ instability were previously described [4, 5]. Ulna styloid fracture and ulnar head subluxation are also associated with DRUJ instability indicating TFCC disruption [6]. The detrimental effects on DRUJ stability increase as ulna styloid fracture goes more proximal [7]. However, controversies exist about whether the ulna styloid fracture is the main reason for DRUJ instability [8]. The ulnar head extensively rotates on the sigmoid notch of the radius in forearm pronation/supination thus fractures of the sigmoid notch alter DRUJ mechanics. Additionally, residual articular incongruity at the radiocarpal and radioulnar joints leads to posttraumatic degenerative changes. Rozental et al. classified sigmoid notch fractures with computed tomography (CT) imaging according to distal radioulnar articular surface extension and fragment displacement [9].

Although the mainstream classifications of DRFs were described on plain radiographs, CT imaging with 3D reconstructions and thinner slices provided more accurate information about fracture extension and stability [10, 11]. Various methods have been described for assessing DRUJ stability, while radioulnar ratio (RUR) measurement on CT images was found as the most sensitive evaluation method described by Mino et al. [12]. Previous studies evaluated the functional outcomes of the sigmoid notch and ulna styloid fracture with DRFs [6, 13]. This study aims to evaluate the effects of the sigmoid notch and ulna styloid fracture types on DRUJ alignment in DRFs following closed reduction and casting by measuring RUR on CT images.

## 2. Materials and methods

Patients admitted to an emergency department of a tertiary care hospital with DRFs between January 2016 and December 2020 were retrospectively evaluated. This study was performed in line with the principles of the Declaration of Helsinki. Approval was granted by the local ethics committee. (02.05.2023/GO23/361). Patient demographics and clinical information were collected through the hospital patient information system (Nucleus Medical Information System). Patients over 18 years of age with a diagnosis of distal radius fracture (ICD 10 code S52.5), patients with available radiographs, and postreduction CT imaging in a neutral forearm position were included in the study. Patients without postreduction CT imaging; patients with open DRFs fractures; patients with a history of neuromuscular diseases, neurologic involvement, previous fracture, or surgery in the ipsilateral extremity were excluded. All measurements were done using Picture Archiving and Communication System.

**Figure 1:**
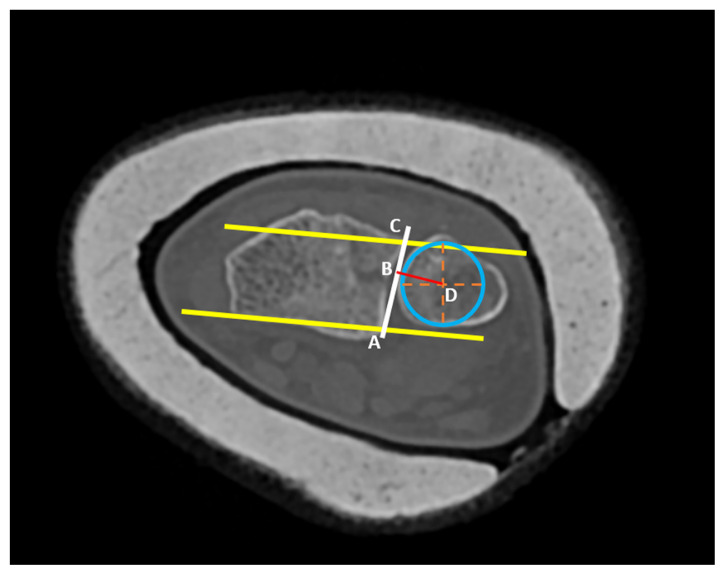
RUR method calculation: A) The lines connecting the radius’s dorsal radial and ulnar borders and connecting the volar radial and ulnar borders are drawn (yellow lines). Then, the line connecting the A (volar side) and C (dorsal side) points, which are the most ulnar points of the drawn lines touching the radius, was drawn. A concentric circle was drawn at the head of the ulna and the line closest to the (AC) line was drawn from the center of this circle (red line). The intersection of these two lines was determined as point B. The length of the line segment AB divided by the length of the line segment AC was determined as the RUR value (AB/AC).

**Figure 2:**
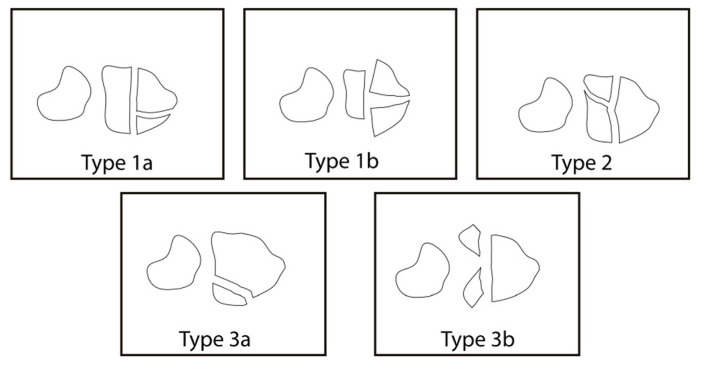
Rozental Classification [14]. Type 1a: Displaced intra-articular DRFs without extension into the sigmoid notch. Type 1b: Separation of the entire sigmoid notch from the remaining fracture fragments. Type 2: Intra-articular DRFs extending into the sigmoid notch, without fragment displacement. Type 3a: Fracture extension into the sigmoid notch with one displaced fracture fragment. Type 3b: Fractures extending into the sigmoid notch with 2 displaced fracture fragment.

Of the 734 patients with distal radius fracture, 202 patients (110 females, 92 males) fulfilled the criteria. All patients with suspected fractures were initially evaluated with standard anteroposterior and lateral wrist radiographs. In our institution, closed reduction and casting are performed for every DRF amenable to closed reduction. Postreduction CT imaging was performed in DRFs extending to DRUJ. The RUR method described by Mino et al. was used to evaluate DRUJ subluxation [14]. The RUR method was preferred as it is a sensitive and practical method for daily clinical use. Mino criteria and RUR measurements were evaluated by estimating appropriate anatomical landmarks. On the axial CT images, the first line was drawn from the dorsal ulnar side corner and dorsal radial side corner of the radius. The second line was drawn from the volar ulnar and radial corners of the radius. RUR was calculated after locating the closest point of the line formed by combining these two lines at the ulnar border of the radius, the ratio of the volar part to the dorsal part of the line is located (Figure 1). The presence of the ulna head between the volar and dorsal lines was considered normal and was chosen as the criteria for subluxation. Sigmoid notch fractures, barely seen in standard radiographs, were classified by Rozental et al. [9]. Sigmoid notch fractures were evaluated for the displacement of the fragments, intra-articular step off, and interfragmentary gapping. According to Rozental’s classification system, nondisplaced intra-articular DRFs without extension into the sigmoid notch were classified as type1a; displaced intra-articular DRFs without extension into the sigmoid notch, but with separation of the entire sigmoid notch from the remaining fracture fragments were classified as type 1b; intra-articular DRFs extending into the sigmoid notch, without fragment displacement were classified as type 2; fractures extension into the sigmoid notch with 1 displaced fracture fragment were classified as type 3a and fractures extending into the sigmoid notch with 2 displaced fracture fragment were classified as type 3b (Figure 2). Radiographic measurements were blindly done by a fellowship-trained trauma surgeon (SK).

**Figure 3:**
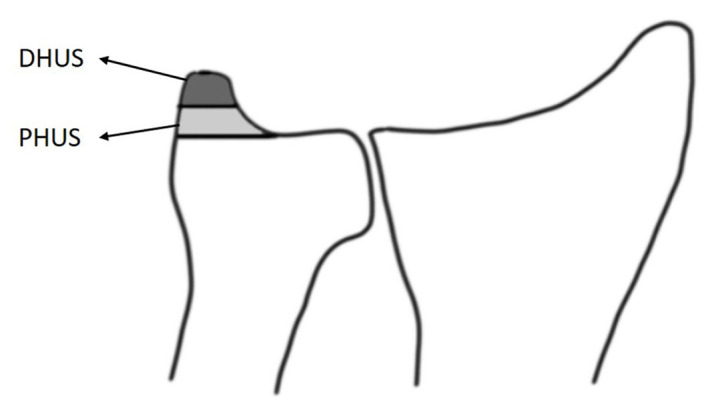
The distal 50% of the ulnar styloid was grouped as Distal Half of Ulnar Styloid (DHUS) and the proximal 50% as Proximal Half of Ulnar Styloid (PHUS) fractures.

For subgroup analysis, DRFs were divided into two groups according to sigmoid notch displacement as nondisplaced in the sigmoid notch fractures (NDS) (Rozental Type 1a and Type 2) and displaced sigmoid notch (DS) fractures (Rozental Type 3a and 3b); ulna styloid fractures were divided into two groups according to height ratio of the fractured ulna styloid as the proximal half ulna styloid (PHUS) and distal half fractures of the styloid (DHUS) (Figure 3). The relationship between PHUS/DHUS fractures and displaced sigmoid notch fractures was evaluated.

The data obtained in the research were evaluated in the software package SPSS (IBM SPSS Statistics for Mac OS, Version 26.0. Armonk, NY). The correlation between RUR values and age was evaluated. The mean age and RUR values were compared in the groups based on Rozental classification, ulnar styloid fracture groups, and whether the fracture involved the DRUJ. Frequency and percentage analysis were used to determine the descriptive characteristics of the patients participating in the study and mean and standard deviation statistics were used to analyze the scale. We scrutinized Kurtosis and Skewness values to assess the normality of the research variables. The prevailing notion in the relevant literature considers results within the range of +1.5 to −1.5 and +2.0 to −2.0 for the kurtosis and skewness values of the variables as indicative of a normal distribution [15, 16]. It was determined that the variables showed normal distribution. Parametric methods were used in the analysis of the data. Relationships between patients’ continuous variables were examined through Pearson correlation analysis. T test, one-way analysis of variance (ANOVA), and post hoc (Tukey, LSD) analyses were used to examine the differences in scale levels according to the descriptive characteristics of the patients. Differences between the ratios of categorical variables in independent groups were analyzed with Chi square and Fisher’s exact tests.

## 3. Results

In the study, 202 patients were included, with 92 (45.5%) being male and 110 (54.5%) female. The mean age of the study patients was 49.9 (SD 15.8) (18–90). Ulna styloid fractures were diagnosed in 84 patients (46.5 %). According to Rozental’s sigmoid notch fractures classification, 86 (42.6%) patients were classified as type 1a, 59 (29.2%) patients were classified as type 2, 43 (21.3%) patients were classified as type 3a and 14 (6.9%) patients were classified as type 3b.

The mean RUR value was 0.55 ± 0.1 (0.23–0.82) among all study patients. The RUR values did not differ according to patients’ sex (p > 0.05). The RUR values of the patients differed significantly according to Rozental’s classification (F = 15.810; p = 0 < 0.05; η^2^ = 0.193) (Table 1). The mean age of the patients showed significant differences according to the sigmoid notch fracture types (F (3, 198) = 4.380; p < 0.05). The mean age of Rozental type 1a was 48.5; type 2 was 51.4; type 3a was 46.5, and type 3b was 62.8 years. The mean age of patients with Rozental type 3b was significantly higher (p < 0.05).

There was a significant difference between mean RUR values of Rozental type 3a and type 1 and 2 patients (p < 0.05) (Table 2). The correlation analyses between age and RUR values showed r = −0.253 negative weak (p < 0.05) correlation.

There was a significant difference in ulna styloid fracture types (PHUS/DHUS) based on the displacement of sigmoid fractures- (χ^2^ = 4.488 p = 0.034). The rate of PHUS fractures associated with DS fractures was higher than DHUS fractures (Table 3).

## 4. Discussion

Residual DRUJ instability is a common complication of DRFs. Because of the complex bony/ligamentous anatomy of DRUJ and the extensive range of motion of the ulna head over the sigmoid notch, recognizing the factors leading to DRUJ instability is challenging. Biomechanical studies on cadavers showed that volar or dorsal angulation of the distal radius alters DRUJ joint kinematics [17, 18]. TFCC is the most critical DRUJ stabilizer consisting of fibrocartilaginous and ligamentous structures [19]. In addition, the pronator quadratus, extensor carpi ulnaris tendon, DRUJ capsule, and interosseous membrane form secondary soft tissue stabilizers [20]. Even though soft tissue factors were described, there is little known about fracture-associated factors contributing to DRUJ instability.

Evaluation of DRUJ bony integrity is as crucial as radiocarpal joint evaluation in DRFs [21]. Rozental et al. findings indicated that CT scanning unveiled an extension of fractures into the sigmoid notch in 65% of DRFs, a contrast to the mere 35% of cases where such extension was discernible solely through plain radiographs [9]. Additionally, in a recent study, fractures extending to the sigmoid notch were found as high as 84.3% [1]. Nakanishi et al. claimed that surgical fixation of the DRUJ fragments might improve DRUJ stability on 3D reconstructed CT images [22]. According to a study in which 139 patients were evaluated, sigmoid notch and proximal ulnar styloid fractures caused significantly lower Gartland and Werley scores [13]. Kong et al. observed that patients with displaced sigmoid notch fracture experienced limitation and pain in forearm rotation compared to those without [23]. In our study, displaced sigmoid notch fractures related to higher RUR values indicated DRUJ subluxation on postreduction CT images. To evaluate the clinical implication of this finding, further clinical and functional evaluation is demanded.

Fractures of the ulna styloid are recognized for their impact on the stability of the DRUJ. Nevertheless, a consensus regarding the correlation between DRUJ instability and ulna styloid fractures remains elusive. In the well-known study of May et al., it was shown that ulna styloid base fractures were more associated with DRUJ instability than smaller distal fragments in patients with DRFs [24]. In a cadaveric study,12 wrist specimens were evaluated with optical motion capture, indicating that ulna styloid base fractures resulted in DRUJ instability in forearm rotation [25]. In another cadaver study in which 8 specimens were examined, all types of ulnar styloid fractures were found to be associated with DRUJ instability [26]. In Kazemian’s study, patients treated with ORIF were evaluated with the modified radioulnar line method on postoperative CT scan, and no statistically significant difference was found between the development of DRUJ instability and ulnar styloid fractures [27]. In our study, the mean RUR values of DRFs with sigmoid notch extension showed no significant difference according to ulna styloid involvement. In addition, there was no significant difference between the mean RUR values according to the size of the ulna styloid fracture.

We found higher patient age with Rozental type 3A fractures compared to Rozental type 1A and type 2, which may be attributed to decreased bone density of distal radius with aging. These results may suggest that fractures associated with DRUJ instability are more likely to occur in the elderly. Functional outcomes following DRFs should be optimized and DRUJ instability should not be overlooked as many geriatric patients remain active in daily life. Tulipan et al. compared functional and radiographic results of high level independent geriatric patients aged between 65 to 74 years and over 75 years with DRFs. They found no significant differences between the age groups. In a study examining 120 wrists using force-monitor ultrasonography, reported that DRUJ stability could change with increasing age and a stiffer wrist could be seen in older patients. Degenerative changes within the stabilizing ligaments of the wrist in addition to decreased flexibility may result in higher force transition to the bones causing a severe injury. In our study, there was a negative low correlation between age and RUR values (r = −0.253; p < 0.05). Given these findings, the surgeon should be aware of the stigmas associated with DRUJ instability in elderly patients with DRFs. Evaluation of RUR in CT scans in elderly patients, especially in fractures extending to the distal radioulnar joint, may be helpful in treatment planning.

Displaced sigmoid fractures were found more frequently with PHUS fractures. Thus, an attempt to assess the integrity of DRUJ should be made considering sigmoid notch fractures are commonly overlooked on standard radiographs. In a recent study, Hruby et al. showed an accuracy of 45.8% for radiographs in diagnosing sigmoid notch fractures [28]. After observing the PHUS fracture, it may be worth considering the evaluation of the wrist with a CT scan, even if the fracture appears well reduced in standard radiographs.

Various radiologic methods were described for assessing DRUJ subluxation [29]. In a study on 12 cadaver wrists in which 3D imaging was performed after creating different instability patterns, Swartmann et al. showed that the RUR method is one of the most sensitive methods to evaluate DRUJ instability [29]. Park et al. examined RUR values were examined in healthy patients [30]. The mean RUR was 0.5 in the forearm neutral position, but average values ranged between 0.32 to 0.69. Given the high degree variations in measurement and position-dependent results, comparing with the contralateral wrist could facilitate better evaluation in these patients. We used the RUR method in this study because it is reliable, easy to teach to residents and observers were familiar with the technique.

There are some limitations of our study. Firstly, this is a retrospective observational study. Tomography views were obtained immediately after the fracture reduction and the treatment process was not evaluated. We were unable to evaluate the treatment outcomes. In addition, CT images of the contralateral wrist were not obtained, so we were unable to do a side-to-side comparison of RUR values. However, this could cause unnecessary radiation exposure for our patients. Since this is a radiological study, the patients were not evaluated for clinical DRUJ instability. The precision of the RUR method in assessing clinical DRUJ instability is on debate. However, we only used the RUR method to assess DRUJ subluxation. Concluding about the relationship between sigmoid/ulna styloid fracture types and clinical DRUJ instability is beyond the scope of our study. Further studies may reveal the relationship between increased RUR and clinical outcomes.

In conclusion, displaced sigmoid notch (DS) fractures and higher patient age are associated with DRUJ subluxation on postreduction CT images following DRF. DS fractures are seen more commonly with PHUS fractures than DHUS. Patients with PHUS should be carefully assessed for sigmoid notch fractures and DRUJ congruency. These findings could be helpful for preoperative decision-making in the treatment of DRFs.

## Figures and Tables

**Table 1:** Comparison between fracture types and radioulnar ratio.

Fracture Groups	n	Radioulnar ratio (RUR) (Mean ± SD)
**Ulna styloid fracture**		
**No fracture**	108 (46.5 %)	0.54 ± 0.08
**1 (0%–25%)**	10 (5.0%)	0.54 ± 0.08
**2 (25%–50%)**	11 (5.4%)	0.55 ± 0.10
**3 (50%–75%)**	20 (9.9%)	0.57 ± 0.12
**4 (75%–100%)**	53 (26.2%)	0.58 ± 0.12
**p=**		0.157
**Rozental**		
**1a**	86 (42.6%)	0.52 ± 0.07
**2**	59 (29.2%)	0.54 ± 0.09
**3a**	43 (21.3%)	0.63 ± 0.10
**3b**	14 (6.9%)	0.57 ± 0.13
**p=**		**<0.00**
		3a > 1, 3a > 2, 3a > 3b (p < 0.05)

**Table 2:** Correlation between sigmoid notch fracture (Rozental) types and patient age.

Rozental	n	Age (Mean ± SD)	p
**1a**	86	48.5 ± 16.8	0.005*
**2**	59	51.4 ± 14.5	
**3a**	43	46.5 ± 12.2	
**3b**	14	62.8 ± 19.2	

SD: Standard deviation

* indicates statistically significant difference between Rozenthal 3b and 1, 3b and 2, 3b and 3a.

**Table 3:** Correlation of ulna styloid and sigmoid notch fracture.

Group		DHUS	PHUS	Total	P
**NDS**	N	15	33	48	0.034
	%	31.3%	68.8%	100%	
**DS**	N	6	40	46	
	%	13%	87%	100%	
**Total**	N	21	73	94	

NDS: Nondisplaced sigmoid notch fracture; DS: Displaced sigmoid notch fracture;

DHUS: Distal half fracture of ulna styloid; PHUS: Proximal half fracture of ulna styloid.
